# Novel Magnetic Elastic Phase-Change Nanodroplets as Dual Mode Contrast Agent for Ultrasound and Magnetic Resonance Imaging

**DOI:** 10.3390/polym14142915

**Published:** 2022-07-19

**Authors:** Ramish Riaz, Hira Waqar, Nasir M. Ahmad, Shah Rukh Abbas

**Affiliations:** 1 Department of Industrial Biotechnology, Atta Ur Rahman School of Applied Biosciences, University of Sciences and Technology, Islamabad 44000, Pakistan; rriaz.phdabs16asab@asab.nust.edu.pk (R.R.); hirawaqar1996@gmail.com (H.W.); 2 Biosensors and Therapeutics Laboratory, School of Interdisciplinary Engineering & Science, National University of Sciences and Technology, Islamabad 44000, Pakistan; 3 Department of Material Engineering, School of Chemical & Materials Engineering, National University of Sciences and Technology, Islamabad 44000, Pakistan; nasir.ahmad@scme.nust.edu.pk

**Keywords:** dual-mode contrast agent, magnetic resonance imaging, ultrasound, iron oxide nanoparticles, PCNDs, poly glycerol sebacate

## Abstract

Recently, dual-mode imaging systems merging magnetic resonance imaging (MRI) and ultrasound (US) have been developed. Designing a dual-mode contrast agent is complex due to different mechanisms of enhancement. Herein, we describe novel phase change nanodroplets (PCNDs) with perfluoropentane encapsulated in a pre-polyglycerol sebacate (pre-PGS) shell loaded with polyethylene glycol (PEG)-coated iron oxide nanoparticles as having a dual-mode contrast agent effect. Iron oxide nanoparticles were prepared via the chemical co-precipitation method and PCNDs were prepared via the solvent displacement technique. PCNDs showed excellent enhancement in the in vitro US much more than Sonovue^®^ microbubbles. Furthermore, they caused a susceptibility effect resulting in a reduction of signal intensity on MRI. An increase in the concentration of nanoparticles caused an increase in the MR contrast effect but a reduction in US intensity. The concentration of nanoparticles in a shell of PCNDs was optimized to obtain a dual-mode contrast effect. Biocompatibility, hemocompatibility, and immunogenicity assays showed that PCNDs were safe and non-immunogenic. Another finding was the dual-mode potential of unloaded PCNDs as T1 MR and US contrast agents. Results suggest the excellent potential of these PCNDs for use as dual-mode contrast agents for both MRI and US.

## 1. Introduction

Medical imaging has long been among the first line of investigation for the diagnosis and prevention of disease. With the advancement in imaging modalities, the use of multiple modalities for disease diagnosis is common. None of the imaging modalities alone can be called ideal as all of them have certain limitations [1]. Computed tomography (CT) and X-rays are cost-effective and take less time; however, they require the use of ionizing radiation. Ultrasound is non-ionizing in real-time, but has limited contrast resolution, while magnetic resonance imaging (MRI) has superb contrast resolution but is limited due to its temporal resolution. The concept of fusion imaging is a recent idea that came to compensate for the limitations of a single imaging modality. Positron emission tomography (PET)/CT and PET/MRI systems are very common fusion imaging modalities. In recent years, dual-mode systems merging MRI and ultrasound have been developed for cardiac and abdominal imaging, especially for image-guided interventions. Both modalities utilize non-ionizing radiations and are complementary in terms of contrast and temporal resolution [2].

Contrast agents in medical imaging are used to enhance the differentiation between tissues, highlighting pathological processes from anatomical ones, perfusion imaging, and functional imaging at a molecular or cellular level [2,3]. In dual-mode applications, the need for a contrast agent varies depending upon the mechanism of contrast enhancement. In ultrasound, the viscoelastic response of the contrast agent inside the acoustic field is the basic mechanism behind contrast enhancement. Core-shell microbubbles are used as contrast agents in ultrasound and their acoustic response strictly depends upon the elasticity of the shell material [4,5,6]. Lipid-based microbubbles (soft shell microbubbles with an elastic modulus less than 30 MPa) are famous for their excellent viscoelastic response in the acoustic field and appear echogenic compared to polymer shell microbubbles. However, lipid-based microbubbles are comparatively less stable than polymeric microbubbles which usually have stiffer shells with high elastic modulus values [4,7,8]. Recently, hybrid shells with double layers have also been proposed to overcome the limitations of both shell types [9,10].

In MRI, contrast enhancement occurs due to the shortening of longitudinal (T1) and transverse (T2) relaxation times. Gadolinium-based contrast agents are currently approved for the T1-weighted positive contrast effect, i.e., an increase in image intensity in regions of contrast uptake, and superparamagnetic iron oxide nanoparticles (SPIONs) for the T2-weighted negative contrast effect, i.e., a decrease in signal intensity upon contrast administration [11].

With the advent of fusion imaging, interest in the development of dual-mode contrast agents for MRI and ultrasound is increasing. However, the design of dual-mode contrast agents is complex due to different mechanisms of enhancement of both modalities. For fabricating a dual-mode contrast agent, nanoparticles are needed to be incorporated into the core or shell of microbubbles. However, the incorporation of nanoparticles increases the stiffness of the shell leading to decreased oscillations in the acoustic field, resulting in low echogenicity or making it suitable only for therapeutic applications involving a high-frequency ultrasound range [2]. For designing a dual-mode contrast agent for a low-frequency ultrasound and MRI, a balance is needed to keep the shell sufficiently elastic with a significant number of nanoparticles to create the MR effect [12,13].

Recently, magnetic liposomes have been used as a dual-mode contrast agent for MRI and ultrasound [12,14]. Among polymeric contrast agents, polyvinyl alcohol-polylactic acid (PVA-PLA) microbubbles filled with nitrogen gas and encapsulating SPIONs have been reported [2]. The research included a study on different parameters and optimized the synthesis to obtain a satisfactory contrast on both modalities. An increase in the density of SPIONs resulted in a reduced contrast on ultrasound due to increased stiffness of the shell. Furthermore, the results showed better enhancement when SPIONs are attached to the shell rather than encapsulated in the core [2]. Recently, Tang et al. has developed perfluorohexane (PFH) core loaded with doxorubicin and Fe_3_O_4_ loaded PLGA microbubbles for theragnostic applications. Since PFH has high boiling point, MBs are responsive for HIFU applications [15]. Another study showed the successful dual-mode potential of lactoferrin conjugated PLLA PCNDs with PFP as the core gas for 10 MHz ultrasound imaging and T2 MRI [16].

Stride et al. studied the effect of gold nanoparticles in the shell of microbubbles and found that the acoustic response of microbubbles increased at lower mechanical index (MI) values due to an increase in backscatter [7]. The phenomenon behind this is predicted to be an increase in non-linear oscillations due to resistance towards compression by closely packed nanoparticles, resulting in expansion-only behavior. However, at high MI values, the increased stiffness leads to dampening of oscillation. Therefore, for the incorporation of nanoparticles, the shell material must be of a very low modulus so that even after the incorporation of nanoparticles, it should be sufficiently elastic to oscillate in the acoustic field and enhance the echogenicity of the resultant image [7,13].

Elastomers are a new class of polymers, known for their high viscoelastic properties, i.e., low elastic modulus and high failure strain. Most of them are applied in the field of tissue engineering as patches for cardiac valves, nerve conduits, and skin grafts. In the field of acoustics, they have been used as sound enhancers in piezoelectric transducers and as phantom material for ultrasound imaging due to their acoustic impedance near body tissues [17,18]. Poly glycerol sebacate (PGS) is a thermoset polymer increasing applied since its discovery in 2002. The material is highly biocompatible, biodegradable, and highly elastic (shear modulus ranging from 0.1 to 1.2 MP a) with low water retention capacity. The elastic properties are dependent upon curing time and temperature. Without curing, the pre-polymer is formed, which is soluble in organic solvents and has a low elastic modulus. Upon curing, the degree of esterification (DE) increases. At 80%, the DE material is insoluble with a higher modulus. Few papers have reported on the drug delivery applications of prepolymer; however, the bulk of the literature largely focuses on tissue engineering applications of the fully cured polymer [19,20]. In our previous work, we synthesized pre-PGS with 56% esterification and theoretically predicted its acoustic response [21]. The results showed pre-PGS to be an excellent shell material giving better oscillations and higher scattering cross-sectional area than Sonovue^®^ microbubbles. The predicted resonance frequency for bubbles with a radius of 1 to 10 µm lies within the diagnostic range, i.e., 1 to 10 MHz [21]. In the current study, we have utilized the pre-PGS polymer as shell material for preparing ‘PCNDs’ by exploiting its elastic properties which match with the reported soft-shelled microbubbles [21]. ‘Phase change nanodroplets (PCNDs)’ fits the term as the liquid PFP core phase converts to an inflated gas core upon exposure to body temperature, i.e., at 37 °C, and also upon ultrasonic exposure due to achievement of acoustic droplet vaporization (ADV) threshold. Since pre-PGS has a low modulus at this stage, we predict that the incorporation of magnetic nanoparticles will not significantly affect its elastic properties and pre-PGS-based PCNDs will serve as an ideal material for a bimodal contrast agent for MRI and ultrasound imaging.

## 2. Materials and Methods

### 2.1. Materials

Sebacic acid (99% purity), glycerol (99% purity), polyethylene glycol (400 Dalton), sodium hydroxide, acetone, span 20, tween 80, calcium chloride (CaCl_2_), Di-mercapto succinic acid (DMSO) and ethanol (99% purity) were purchased from Sigma Aldrich (Karachi, Pakistan). Ferric chloride hexahydrate (FeCl_3_·6H_2_O) and ferrous chloride tetrahydrate (FeCl_2_·4H_2_O) were purchased from DUKSAN (Gyeonggi-do, South Korea). Perfluoropentane was purchased from Shanghai Tianfu (Shanghai, China). PBS tablets were purchased from Oxoid (Basingstoke, UK). The thrombin reagent was purchased from Roche Pharma (Karachi, Pakistan). Gadovist was purchased from Bayer’s Pharmaceuticals (Karachi, Pakistan). Sonovue^®^ (Bracco Int. High Wycombe, UK) was purchased from Shifa International Hospital Pharmacy (Islamabad, Pakistan). The ELISA kit was purchased by Nanjing Pars Biochem Ltd. (Nanjing, China) The kit contained standards, antibody-coated microtiter plate, chromogen A, chromogen B, HRP conjugate and stop solution.

Blood was collected from healthy donors in citrated and EDTA-containing vials. Vials and Aspirin (High noon Laboratories Ltd., Lahore, Pakistan) were purchased from a local pharmacy. LPS extracted from E. coli was a gift from the National Institute of Health, Islamabad, Pakistan. MCF-7 cell lines (HTB-22™) were purchased from ATCC (Manassas, VA, USA).

### 2.2. Methods

#### 2.2.1. Synthesis of Pre-PGS

Pre-PGS was synthesized via melt condensation reaction reported in the literature [21,22]. Briefly, an equimolar concentration (1:1) of glycerol and sebacic acid was taken and heated for 15 min to ensure homogeneity. Afterward, the reaction proceeded under continuous nitrogen flow at 180 °C. A yellowish viscous sticky elastomer was obtained which converted to a waxy solid upon cooling. Synthesis was confirmed by the presence of an ester bond upon FTIR.

#### 2.2.2. Synthesis of PEG Coated SPIONS

Iron oxide nanoparticles were prepared via the chemical co-precipitation method. Briefly, iron precursor solutions were prepared by dissolving iron chlorides in deionized water. The ferric chloride (FeCl_3_) and ferrous chloride (FeCl_2_) solutions in a stoichiometric ratio of 2:1 were stirred at 800 rpm at 80 °C under an inert atmosphere. Precipitation was carried out by dropwise addition of sodium hydroxide solution. The reaction proceeded for 30 min. The change of color to black indicated the formation of iron oxide nanoparticles. In total, 1 mL of PEG was added and the reaction was further continued for 1 h to coat the nanoparticles. The prepared particles were decantated and washed with acetone and water followed by heating in a drying oven at 60 °C.

#### 2.2.3. Preparation of Unloaded and Loaded PCNDs

PCNDs were prepared via the solvent displacement method. For the synthesis of unloaded PCNDs (without nanoparticles), the pre-polymer was dissolved in the organic phase (ethanol) with a span of 20. PFP emulsion (5%) was prepared by dropwise addition of PFP in an aqueous phase with tween 80 under probe sonication. Sonication was performed via Hielscher Ultrasonicator (UP400S) at 120 W for 10 min with a 1 min cycle of sonication preceded by a 1 min break upon ice bath. The aqueous phase was extruded via a 0.4-micron filter and added dropwise into an organic phase. For solvent evaporation solution was stirred for 3 h at room temperature. Synthesized unloaded PCNDs were washed three times via centrifugation at 2000 rpm. For the synthesis of loaded PCNDs, PEG-SPIONs were incorporated in both the shell and core of PCNDs. For incorporation of PEG-SPIONs in the shell, they were added in pre-PGS and heated at 120 °C for 10 min. Successful incorporation of nanoparticles in pre-PGS was indicated by a change of white-colored pre-PGS to brown without any residual particles. The SPION-loaded pre-PGS were dissolved in ethanol with a span of 20 and used as an organic phase. The rest of the procedure was the same. For the incorporation of nanoparticles in the core of PCNDs, nano-particles were added to the PFP emulsion during the sonication step. This nanoparticle-containing emulsion was extruded and then added dropwise to the organic phase with pre-PGS. The rest of the procedure was the same.

### 2.3. Characterizations of Polymeric PCNDs

#### 2.3.1. Chemical Characterization

##### Fourier Transform Infrared Spectrometry

For analysis of functional groups in all constructs, Fourier transform infrared spectrometry (FTIR) was performed. The potassium bromide disc method was used and the spectrum was taken at a resolution of 4 cm^−1^ in the range of wavenumber (450–4000 cm^−1^). The spectrum was acquired using a Spectrum-100 (Perkin Elmer, Waltham, MA, USA).

##### Contact Angle Measurements

The water contact angle measurements were performed by sessile drop technique by placing 3 µL of ultra-pure water on a glass slide evenly spread with samples. Measurements were taken via Fibro DAT 1100 (Stockholm, Sweden). The angle between the liquid/vapor interface and the solid/liquid interface (contact angle) was measured by using the ADVANCE software installed in the drop shape analyzer.

##### X-Ray Diffractometry

XRD analysis was performed via XRD, STOE, Darmstadt, Germany (Theta-Theta S/N 65022) with Cu-Kα (λ = 1.54) with 2θ ranging from 0 to 90°.

#### 2.3.2. Confirmation of PFP Encapsulation

##### Encapsulation Efficiency

The encapsulation efficiency of PFP was calculated via UV-visible spectrophotometry (Analytik Jena, Bucha, Germany, Model: SPECORD 200 Plus) at a wavelength of 270 nm. Ice-chilled 75:15 methanol:water was used as a blank. For releasing PFP, the PCNDs pellet was suspended in the ice-chilled methanol:water and centrifuged at 4000 rpm. This results in the disruption of PCNDs with a PFP release in solution and pre-PGS settling at the bottom. For calculation of exact quantity, a calibration curve of PFP was drawn by taking different concentrations of PFP in the ice-chilled methanol: water. The absorption of the sample was compared with the calibration curve of PFP. The encapsulation efficiency of PFP in PCNDs was calculated by the following formula:

$$EE\;\left(\%\right)=\frac{Wi}{Wt}\times 100\;\left(\%\right)$$

where *EE* is encapsulation efficiency, *Wi* is the amount of PFP in solution and *Wt* is the amount of PFP added initially.

##### Optical and Fluorescent Microscopy

Both phase-converted and non-converted PCNDs were visualized under an optical microscope. PCNDs counting was performed via a hemocytometer grid. For phase conversion, PCNDs were first heated at 35 °C and then drop was placed on a slide and set on stage for microscopic visualization.

To confirm the core-shell morphology core, PFP was mixed with fluorescein with an excitation wavelength of 475–490 nm while pre-PGS was mixed with crystal violet dye with an excitation wavelength of 550 nm. The rest of the methodology for synthesis was the same as mentioned above. A drop of PCNDs was visualized and captured via an XDY-2 inverted fluorescence microscope.

### 2.4. Morphological Analysis

#### 2.4.1. Scanning Electron Microscopy

For size and morphological analysis of the nanoparticles and PCNDs, scanning electron microscopy was performed using SEM-JEOL (JSM-6490L, Tokyo, Japan). Samples were diluted in PBS and a drop was placed on a 1 cm × 1 cm glass slide. The slide was dried in a vacuum desiccator followed by sputtering with gold. Images were taken with an accelerating voltage of 10 kV at different magnifications. Size and roughness measurements were performed via the ImageJ software.

#### 2.4.2. Dynamic Light Scattering Analysis

Dynamic light scattering (DLS) analysis of nanoparticles and PCNDs was performed via a zeta size analyzer (Malvern Zeta Sizer ver 7.12, Worcestershire, UK, Serial no MAL1168467) to obtain an average size distribution, polydispersity index, and zeta potential (ζ), i.e., surface charge. Samples were prepared by diluting in PBS followed by pipetting into a plastic cuvette and measured using a zeta analyzer at room temperature.

### 2.5. Acoustic and Magnetic Characterization

#### 2.5.1. Vibrating Sample Magnetometry

The magnetic properties of PEG-coated iron oxide nanoparticles were studied via a vibrating sample magnetometer (VSM) (Cryogenic Ltd., London, UK) at room temperature. For VSM measurements, powder samples were taken in the sample holder and the external magnetic field was changed between −10 and +10 kOe. The hysteresis loop was taken at a frequency of 40 Hz with an amplitude of 3 mm and a phase shift of 123°.

#### 2.5.2. In Vitro Magnetic Resonance Imaging

For checking the contrast effect of T2 weighted images on MRI, an agar phantom was made containing falcons of PCNDs loaded with different concentrations of PEG-coated SPIONs. For comparison with control, Gadovist at high concentration was used to obtain a negative contrast effect on T2. MRI was performed in a 0.3 T machine with a time to repeat (TR) of 5450 ms, time to echo (TE) of 117 ms, window width of 1024, window level of 456 and slice thickness of 5.60 mm. Image intensities were measured using a Micro DICOM viewer. Measurements were taken thrice; mean and standard deviations were then calculated. ANOVA analysis was performed to check the significance between positive and negative control and constructs. Tests were performed in GraphPad Prism version 8. P-value was taken significantly at 0.05.

#### 2.5.3. In Vitro Ultrasound Analysis

The contrast effect of unloaded and loaded PCNDs with different concentrations of PEG-coated SPIONs was checked via in vitro ultrasound imaging (TOSHIBA Applio 500, Kawasaki, Japan) using a 3.5 MHz curvilinear transducer with an MI range from 0.1 to 1.5. The PCNDs number density was determined via a hemocytometer used for cell counting under a light microscope (Optika). In total, 1 × 10^6^ PCNDs were added to 1000 mL of water. Sonovue^®^ MBs were used as positive control while water served as a negative control. The change in intensity was measured via MATLAB code. Measurements were taken thrice; mean and standard deviations were then calculated.

### 2.6. Stability Testing

For estimating the biodegradability profile of 1 g pre-PGS based PCNDs at blood pH and temperature, PCNDs were incubated in PBS at 37 °C After every 3 days, samples were centrifuged and weighed. For estimating the shelf life of both vaporized and non-vaporized PCNDs, ultrasound analysis was performed to check for any change in the contrast effect.

### 2.7. In Vitro Safety Assays

#### 2.7.1. Hemolysis Assay

To establish the safety profile of contrast agents on red blood cells (RBCs), a hemolysis test was performed. Briefly, 3 mL of ethylene diamine tetra acetic acid (EDTA)-stabilized human blood samples were centrifuged at 6000 rpm for 10 min to remove the buffy coat and plasma. The residual red blood cells (RBCs) were washed five times with 3 mL of isotonic PBS to remove the traces of plasma. Washed RBCs were suspended in PBS. Unloaded PCNDs and PCNDs loaded with 10 mg of PEG-coated SPIONs were then diluted in PBS to obtain the PCNDs number densities of 1 × 10^7^, 1 × 10^6^, 1 × 10^5^, 1 × 10^4^, and 1 × 10^3^ PCND.mL^−1^ of PBS. In total, 20 μL of the sample was incubated with 180 μL of the diluted blood cell suspension for 30 min at 37 °C with agitation. Samples were then centrifuged at 1500 rpm. Followed by dilution of supernatant in 9:1 PBS: supernatant. Optical density (*OD*) of diluted supernatant was taken at 550 nm. Triton X-100 (0.1%) was taken as the positive control and PBS as the negative control. The experiment was run in triplicate. The percentage of hemolysis was calculated from mean optical densities by the following formula:

$$Hemolysis\;\left(\%\right)=\frac{\;ODs-ODnc}{ODpc-ODnc}\times 100\;\left(\%\right)$$

where sample *OD* is represented as ODs, negative control *OD* as ODnc and positive control *OD* as ODpc.

#### 2.7.2. Thrombin Time

For thrombin time measurements, citrate blood was taken. Platelet-rich plasma (PRP) was obtained by centrifugation at 2000 rpm for 10 min. PRP was incubated and unloaded with 10 mg PEG-coated SPIONS and loaded PCNDs with number densities of 1 × 10^7^, 1 × 10^5^ and 1 × 10^3^ PCNDs.mL^−1^ at 37 °C for 10 min. Aspirin was taken as positive control while PRP and PRP with PBS were taken as negative controls. The thrombin reagent was then added and the time taken for the plasma to clot was measured.

#### 2.7.3. Plasma Recalcification Time

Blood was collected in sodium citrate blue vials. Firstly, platelet-rich plasma (PRP) was separated by centrifugation of blood at 2000 rpm for 10 min. Then, PRP was further centrifuged for 5 min at 3000 rpm to obtain platelet-poor plasma (PPP). In total, 40 µL of different concentrations of microbubbles were incubated with 100 µL of PPP for 10 min at 37 °C. Aspirin was taken as positive control while PPP and PPP with PBS were taken as negative controls. Clotting was induced by the addition of 20 µL 0.16 M CaCl_2_. Time taken in the formation of thread-like structures was noted.

#### 2.7.4. Biocompatibility Assay

For the MTT assay, MCF-7 cell lines were cultured and plated in 96 well plates followed by incubation for 24 h. Afterward, 200 µL of different concentrations were added to the wells and incubated for 24 h. The plate was then injected with 15 µL of prepared MTT and incubated for 3 h at 37 °C. MTT was pipetted out and 150 µL of DMSO was added to every well followed by incubation at room temperature. The plate was read by an OD reader and measurement of the absorbance was carried out at 550 nm wavelength after 24 h. The experiment was run in triplicate.

#### 2.7.5. Immunogenicity Test

To assess complement activation in response to PCNDs, complement factor 5b (C5b) ELISA was performed via an ELISA kit purchased by Nanjing Pars Biochem LTD. Briefly, blood was collected from two donors in serum gold cap vacutainers with a clot activator and gel separator. Blood was allowed to stand for 20 min followed by centrifugation at 3500 rpm for 10 min. Serum was carefully collected and stored at −20 for an hour. Afterward, serum was incubated with PCNDs at different concentrations, positive and negative control for 45 min at 37 °C to trigger an immune response. PCNDs were serially diluted to obtain the number densities of 1 × 10^7^, 1 × 10^6^, 1 × 10^5^, 1 × 10^4^, and 1 × 10^3^ PCNDs.mL^−1^ For positive control, lipopolysaccharide (LPS) from *E. coli* was used, as it is a potent activator of the complement system. PBS was used as the negative control. For ELISA, briefly, samples, standards and positive and negative controls were added to C5b antibody-coated microtiter plate. Blanks were set separately and the plate was incubated for 30 min at 37 °C followed by washing with a wash buffer 5 times. HRP conjugate was then added and incubated again for 30 min at 37 °C followed by washing with wash buffer 5 times. Chromogen A and Chromogen B solutions were then added and placed in the dark for 15 min at 37 °C. The stop solution was then added and the plate was read under ELISA Plate Reader at 450 nm.

## 3. Results and Discussion

### 3.1. Chemical Characterization of Polymeric PCNDs

Dark brownish-black colored iron-oxide nanoparticles were prepared as a result of the coprecipitation technique. PEG-coated particles were a little lighter, i.e., brown. Magnetic properties of particles were confirmed via placing a simple bar magnet and decanting them. Chemical composition was further confirmed via FTIR analysis. Bare nanoparticles showed a characteristic peak of Fe-O bond at 630, 795, and 880 cm^−1^. The same peaks can be observed in PEG-coated iron oxide nanoparticles but with reduced intensity. These peaks are consistent with peaks reported in the literature [23].

**Figure 1:**
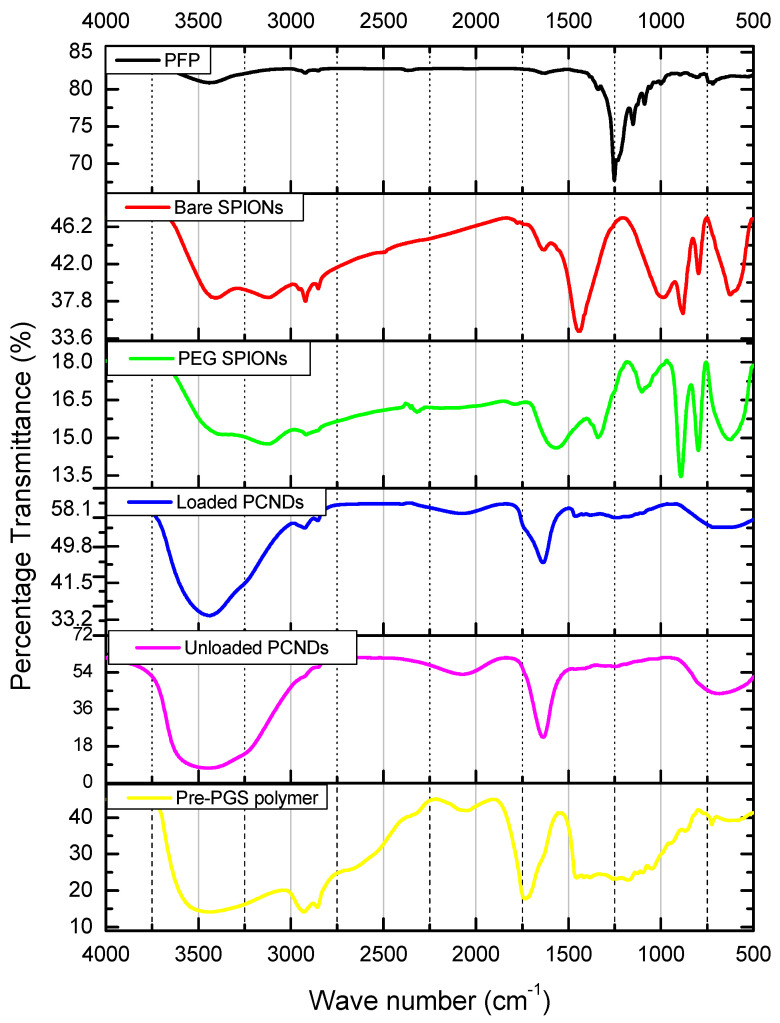
FTIR spectrum of PFP, bare iron oxide nanoparticles, PEG-coated iron oxide nanoparticles, unloaded PCNDs, PEG-coated SPION-loaded PCNDs and pre-PGS polymer. Loading of iron oxide nanoparticles was confirmed via a broad peak at 650 cm^−1^ that encompasses a characteristic Fe-O peak at 630 cm^−1^.

Unloaded PCNDs were white, just like the pre-polymer, while loaded PCNDs had a slight brown color. Pre-PGS polymer showed a characteristic ester peak at 1734 cm^−1^. The formation of unloaded PCNDs shifted the ester peak towards the lower wavenumber due to bounding with surfactants. Pre-PGS PCNDs showed C-F bond vibration between 1230 and 1460 cm^−1^, signifying the encapsulation of perfluoropentane inside the PCNDs. Loaded PCNDs showed similar peaks of unloaded microbubbles at 3440 cm^−1^ and 1631 cm^−1^, but showed a broad peak at 650 cm^−1^, encompassing a characteristic Fe-O peak at 630 cm^−1^. The peak at 2920 was consistently seen in iron oxide nanoparticles, PEG-coated iron oxide nanoparticles and PEG-coated SPIONs PCNDs, but not in unloaded PCNDs. Figure 1 shows the FTIR spectrum of nanoparticles and PCNDs while Table 1 summarizes the observed peaks.

**Figure 2:**
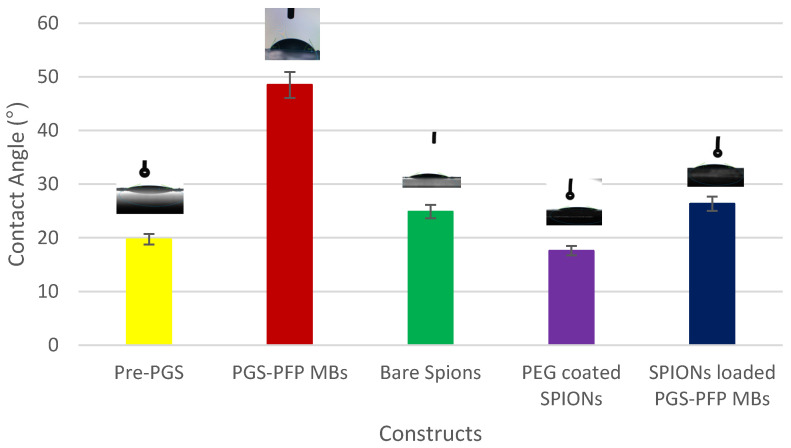
Water contact angle measurements of the pre-PGS polymer, unloaded PCNDs, bare SPIONs, PEG-coated SPIONs and PEG-coated SPION-loaded PCNDs.

The water contact angle measurements of the synthesized nanoparticles are shown in Figure 2. The pre-polymer showed hydrophilic behavior with a water contact angle of 19.7°. Due to the encapsulation of hydrophobic perfluoropentane inside the synthesized PCNDs, the water contact angle increased up to 48.49°. Since PEG is hydrophilic material, PEG-coated SPIONs showed a water contact angle of 17.58°. The incorporation of these SPIONs into PCNDs resulted in a final contact angle of the construct of 26.33°.

Figure 2 shows water contact angle measurements of pre-PGS polymer, unloaded PCNDs, bare SPIONs, PEG-coated SPIONs and PEG-coated SPION-loaded PCNDs.

**Figure 3:**
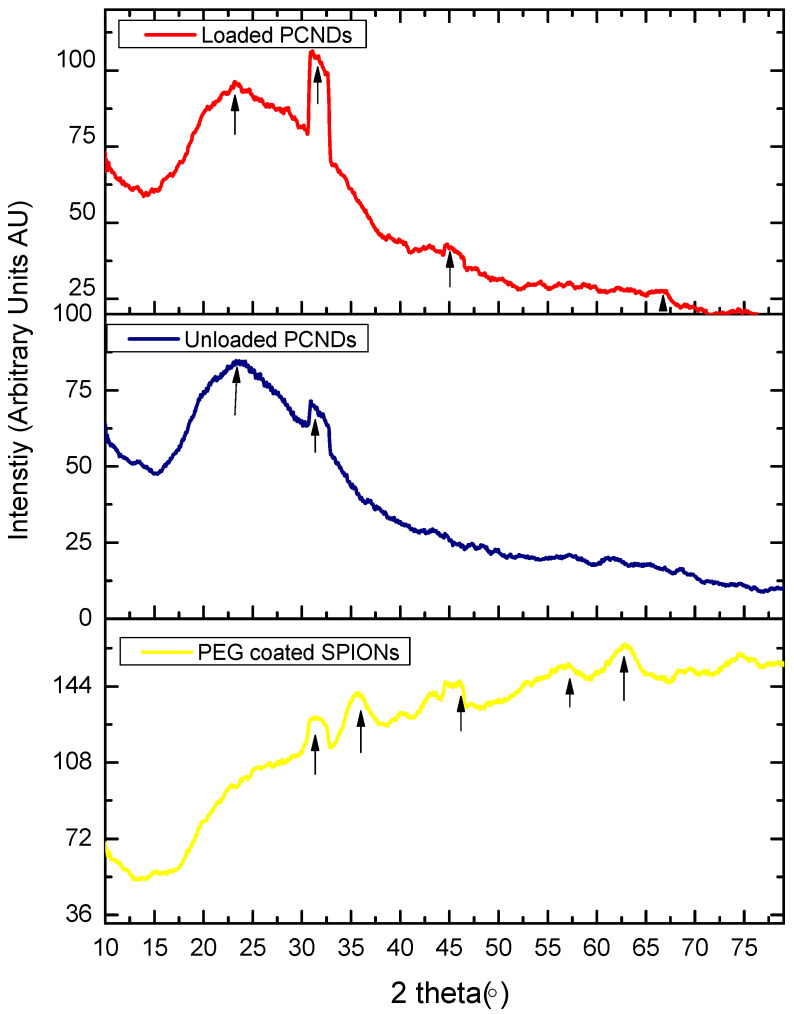
XRD curves of PEG SPIONs, unloaded PCNDs and SPION-loaded PCNDs. PEG SPIONs showed 2θ peaks of magnetite nanoparticles.

Figure 3 shows the X-ray diffractometry analysis of PEG-coated SPIONs, unloaded PCNDs and SPION-loaded PCNDs. PEG-coated SPIONs showed 2θ peaks at 31.35°, 35.35°, 45.1°, 57.15°, 63.07° and 74.5°. The peaks are consistent with peaks of magnetite in the literature matching with the Joint Committee on Powder Diffraction Standards (JCPDS) 19–0629 [33]. Unloaded PCNDs showed a characteristic broad peak between 15° and 35° of PGS polymer center at 23.5° similar to peaks reported for the PGS elastomer in the literature [22,34] (Table 2). Loaded PCNDs also showed a pre-PGS peak along with PEG-SPIONs peaks.

### 3.2. Confirmation of PFP Encapsulation

**Figure 4:**
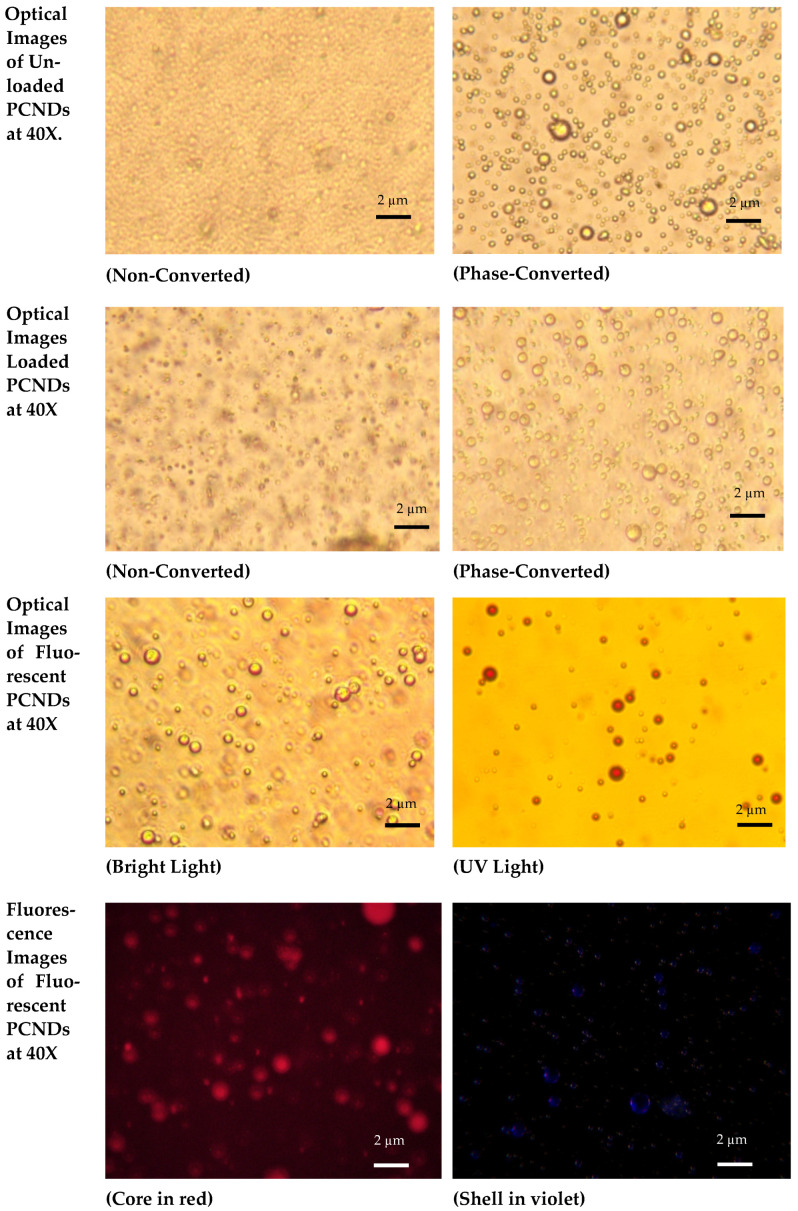
Optical and fluorescence microscopy images of unloaded PCNDs, Loaded PCNDs and fluorescent PCNDs.

Encapsulation efficiency of PFP was found to be 52.6 % (Supplementary Figure S1). Both light microscopic images and fluorescent microscopic images of PCNDs showed a core-shell morphology and phase-conversion property. The PCND’s size increased 5–20 times upon phase conversion. Fluorescence images showed a red core upon excitation at 490 nm and a blue-violet shell upon excitation at 550 nm (Figure 4).

### 3.3. Morphological Analysis

**Figure 5:**
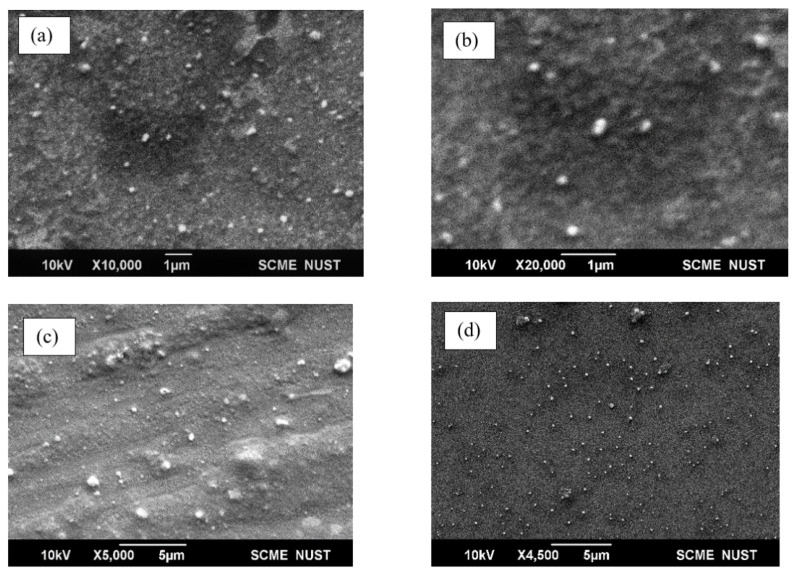
SEM images of PEG-coated SPIONs (**a**) and (**b**) at low and high resolution, loaded PCNDs (**c**) and unloaded PCNDs (**d**).

Figure 5 shows SEM images of PEG-coated SPIONs and PEG-coated SPION-loaded PCNDs and unloaded PCNDs. PEG-coated iron oxide nanoparticles were smooth and spherical upon SEM. The PCNDs were also smooth and spherical with an average size of 132 nm. The incorporation of PEG-coated nanoparticles increased its size and roughness (Figure 5). Supplementary Figure S2 and Table S1 show the surface plots of constructs and roughness measurements, respectively. Particle sizes in electron microscopy are usually smaller than measured by dynamic light scattering due to dehydration under vacuum; DLS also measures hydrodynamic diameter rather than particle size [37].

The hydrodynamic diameter of PEG-coated SPIONs was found to be 190.1 nm. DLS of liquid perfluorocarbons near their boiling point always results in polydispersity due to the presence of both phase converted (larger size) and non-phase converted PCNDs (smaller size) depending upon their boiling point [38]. In the current study, DLS was performed at 25 °C near the boiling point of liquid PFP; therefore, PCNDs showed both phase-converted and non-phase-converted microbubbles. Upon phase conversion, gas bubbles increase in size by 3–10 times. A similar trend has been observed in our results (Table 3). The unloaded PCNDs showed three peaks: peak one with 67.1% intensity was attributed to non-phase converted PCNDs with a size of 210.3 nm while peak 2 and peak 3 with 27.3% and 5.1% intensity corresponded to phase-converted PCNDs at 25 °C. The PEG-coated SPION-loaded PCNDs showed two peaks: one corresponding to non-phase converted PCNDs and the other of phase converted PCNDs. Non-phase converted PCNDs showed a size of 163 nm. Upon phase conversion, it increased to 927.3 nm (Table 3). Studies show that the acoustic droplet vaporization threshold needed for the phase transition of perfluoropentane droplets is achieved either by heating at 29 °C or by providing ultrasound pressure. The incorporation of nanoparticles also helps in reducing the threshold for ADV [39]. Since DLS was performed at 25 °C, the majority (62.6%) of the loaded PCNDs were phase converted while 37.4% remained in a liquid state. However, the majority (67.6%) of unloaded PCNDs were non-phase converted due to the high threshold of ADV. A small peak (5.1%) was observed at 5359 ± 341.6 nm. This can be attributed to droplets’ coalescence. Droplet coalescence is an unavoidable phenomenon that occurs during the process of emulsification due to interfacial tension between dispersed and continuous phases. This can be minimized by using surfactants [40,41]. In this study, tween-80 and span-20 were added during synthesis to minimize this phenomenon.

Colloidal stability of nanoformulations is determined via zeta potential measurements. Zeta potential values of −30 to +30 mV are considered stable resulting in less agglomeration [42]. The surface charge of unloaded PCNDs was found to be −20.5 ± 4.2 mV which was in accord with another study in which pre-PGS nanoparticles were used as drug carriers [22]. PEG-coated SPIONs showed zeta potential equivalent to +32.3 ± 6.6 mV which was similar to other studies focused on PEG-coated SPIONs [43,44]. Upon loading of positively charged SPIONs in the shell of negatively charge PCNDs, the surface charge became −11.9 ± 5.3mV which validates SPIONs incorporation on the surface (Table 3).

### 3.4. Magnetic Characterization of PEG Coated SPIONs

**Figure 6:**
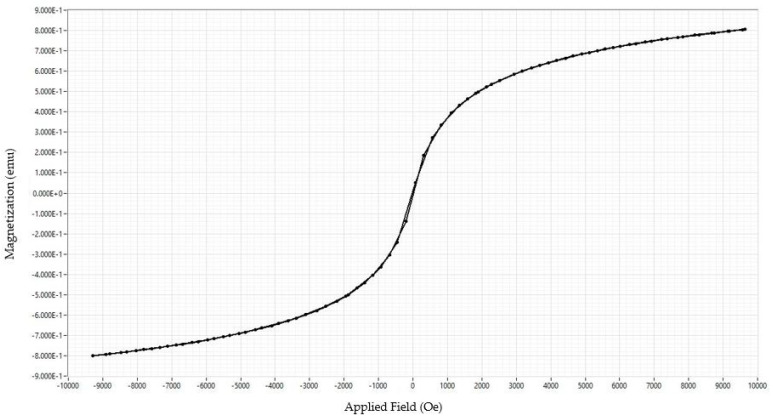
Hysteresis graph of PEG-coated iron oxide nanoparticles showing a characteristic narrow loop of superparamagnetic iron oxide nanoparticles.

For magnetic characterization of synthesized PEG-coated superparamagnetic iron oxide nanoparticles (SPIONs), vibrating sample magnetometry was performed. Figure 6 shows the hysteresis curve of VSM. The absence of a hysteresis loop is indicative of the superparamagnetic behavior of nanoparticles which was further confirmed by near-zero values of remanence magnetization (Mr = 0.125 emu/g) and squareness ratio (Mr/Ms = 0.01). The results were in accordance with other studies, where PEG-coated nanoparticles (less than 50 nm) showed saturation magnetization (Ms) of 23 emu/g with Mr values less than 2 nm [45,46]. Another study with a similar size distribution of PEG-coated nanoparticles (50–300 nm) also showed superparamagnetic behavior with a coercivity value of 27 Oe and an Mr of 2.2 [47]. The current study with a particle size of 190 nm showed a coercivity of 19.06 Oe and an Mr of 0.125 emu/g.

### 3.5. In Vitro MR Imaging

**Figure 7:**
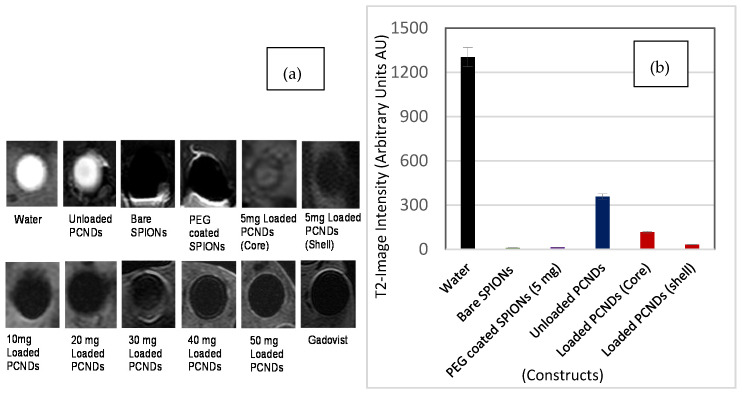
MR image intensity-based analysis of different concentrations of loaded PCNDs: (**a**) shows T2 weighted images (**b**) shows image intensity of constructs.

SPIONs are famous for their ability to reduce transverse relaxation time (T2) by causing increased dephasing between proton spins. SPIONs can be incorporated in both the shell or core of a microbubble for multi-modal properties [2]. Incorporation of SPIONs in the core helps in achieving early ADV, thereby increasing acoustic response and suppressing MR response [39]. While incorporation in the shell increases, the stiffness of the shell results in a decreased acoustic response but strong magnetic field inhomogeneity, thereby increased the dephasing and potent T2 contrast effect [48]. To obtain the contrast effect, there is a need to create balance. In the first step of this study, 5 mg SPIONs were loaded in both the core and shell of PCNDs, and the results were compared. A significant decrease in T2 intensity was noted in shell incorporated PCNDs compared to core ones (Figure 7a). An increase in the concentration of iron oxide particles in the shell increased the susceptibility effect, hence the signal loss (Figure 7b). The results were compared with commercially available Gadovist^®^, predominantly a T1 contrast agent due to the non-availability of Resovist^®^ (T2 contrast agent). Since gadolinium did not affect T2 contrast intensity at low concentration, a high concentration of Gadovist^®^ was used to obtain the T2-susceptibility effects. The results showed comparable response and intensity dropped from 1300 to 20 AU, which in itself depicts a high T2 susceptibility. A significant difference (P < 0.0001) was found between the mean intensity of loaded construct and water (NC) while no difference was found between PEG SPIONs (PC) and loaded PCNDs (P-Value = 0.5318) (Supplementary Table S2).

Different studies are focused on developing magnetic microbubbles and optimizing the concentration to obtain the dual modal response. The majority of the work focuses on magnetic liposomes [49,50,51,52], while few studies use polymer-based microbubbles for loading iron oxide nanoparticles. Among the most commonly used polymers are PVA and PLA. Mostly polymer-based microbubbles respond at a high-frequency range upon ultrasound due to stiff shells [2,16,53,54]. Brismar et al. synthesize incorporated salinized SPIONs and loaded them in 2% PVA solution and achieved a contrast effect in both ultrasound (5 MHz) and MRI (3 T) [12].

For T1 contrast enhancement (increased T1 weighted image intensity), the contrast agent should be capable of decreasing longitudinal relaxation time. This is effectively carried out by paramagnetic agents. Superparamagnetic agents have very little effect on T1 intensity. The materials which have a short T1 time can also be used as T1 contrast agents. Recently, copper oxide (CuO) nanoparticles have been reported to be T1 shortening agents. The particles were also able to speed up the ultrasonic wave and carry the potential of dual-modal imaging [55]. Cationic Fe (III) has also been found to decrease T1 relaxation time [56]. Yoon et al. reported Fe (III)-melanin microbubbles as T1 MR and ultrasound contrast agents [57].

**Figure 8:**
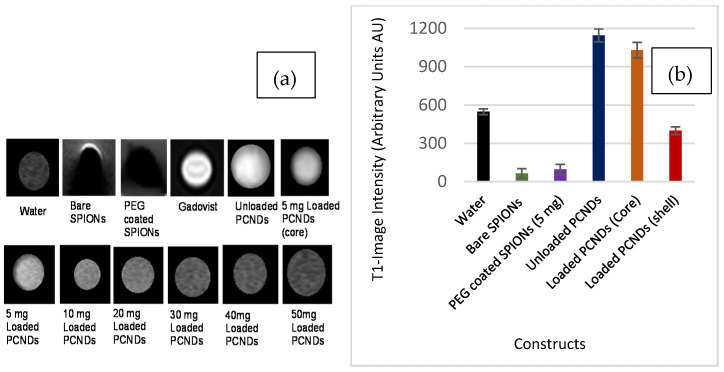
MR image intensity-based analysis of different concentrations of loaded PCNDs. (**a**) shows T1 weighted images (**b**) shows T1 image intensity graphs of the constructs.

An interesting effect was noted in this study. The unloaded PCNDs, i.e., those constructed without any iron oxide nanoparticles, appeared bright both on T1 and T2 weighted images (Figure 7 and Figure 8). A significant difference (P < 0.004) was found between the mean intensity of the unloaded construct and water (NC) (Supplementary Table S3). The appearance was similar to fats with a short relaxation time on both T1 and T2 due to disruption of J coupling upon T2 on spin-echo (SE sequences). This could be due to the internal chemistry of our construct. Pre-polyglycerol sebacate is a polyester formed as a result of polycondensation of glycerol and sebacic acid. Therefore, the material possesses the properties of fatty substances. The property can be exploited for the T1 contrast effect if used alone.

The incorporation of nanoparticles changed the spin–lattice interaction, therefore resulting in a reduction of signal intensity. Techniques optimizing the concentration of iron oxide or chelating with other materials can help make an agent capable of shortening both T1 and T2 relaxation times. Recently, hyperbranched polyglycerol has been successfully conjugated with Gd to make a T1 contrast agent [58]. In another study, mixed polymeric micelles made up of polycaprolactone, polyglycerol, and polyethylene glycol with Gd DOTA and folic acid have also shown promising results as a T1 contrast agent in in vivo experiments [59]. Since the study was focused on the development of multimodal agents for ultrasound and T2 weighted application, the concentration of unloaded PCNDs was not studied in detail. However, current results showed the great potential of these agents to be used in the future.

### 3.6. In Vitro Ultrasound Imaging

**Figure 9:**
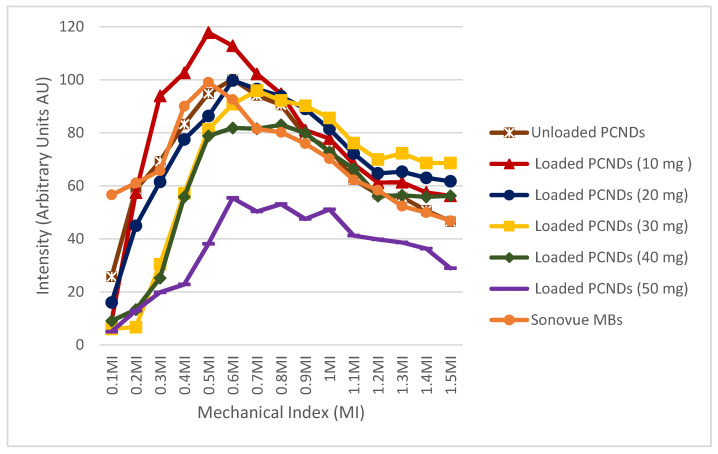
Ultrasound image intensity analysis of different concentrations of PEG-coated SPIONs loaded PCNDs. In total, 10 mg loaded PCNDs showed the best response followed by unloaded PCNDs within the diagnostic MI range.

For characterization of acoustic enhancement by magnetic microbubbles, in vitro ultrasound imaging was performed. Images were taken with different concentrations of PEG-coated SPIONs to get an idea about the change in shell characteristics, i.e., elasticity upon the incorporation of iron oxide nanoparticles. Unloaded PCNDs showed 100 times more enhancement at almost all MI values, which shows its excellent potential to be used as an ultrasound contrast agent. The incorporation of 10 mg PEG-coated SPIONs increased the image intensity up to 120 times. This could be due to an increase in non-linear oscillation or an increase in backscatter upon the incorporation of iron oxide nanoparticles [7]. However further increase in iron oxide nanoparticle concentration resulted in increased damping due to an increase in stiffness of the shell, thereby reducing contrast levels. The highest damping was noted for 50 mg concentration at all MI values. It was also observed that an increase in nanoparticle concentration also increased the stability of PCNDs as observed by an increase in image intensity at high MI (0.9 to 1.5) by an increase in SPIONs concentration within the FDA-allowed range for microbubble imaging, i.e., 0.1 to 0.9 MI. The highest enhancement levels were shown by 10 mg concentration; therefore, PCNDs loaded with 10 mg concentration were considered as optimum. Maximum backscatter was observed at 0.5 MI, i.e., medium MI range. An almost 120% increase in echogenicity was observed with 10 mg SPION-loaded PCNDs. The intensity was higher than the standard Sonovue^®^ microbubbles. Figure 9 shows detailed curves of different iron oxide concentrations at a 3.5 MHz frequency and complete MI range (0.1 to 1.5). The negative control intensity was subtracted to obtain a normalized intensity in intensity curves.

**Figure 10:**
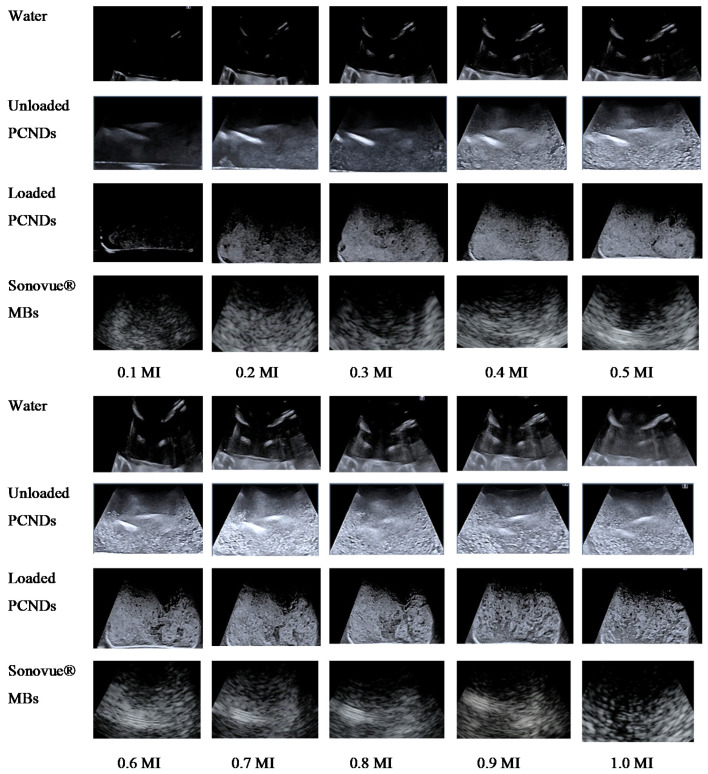
Ultrasound images of water (negative control), unloaded PCNDs, 10 mg PEG-coated SPION-loaded PCNDs in comparison to Sonovue^®^ as a positive control at MI (0.1 to 1) on 3.5 MHz frequency.

The majority of dual-mode contrast studies are focused on high-frequency applications, while most abdominal and cardiac examination involves 3.5 MHz frequency ultrasound [15,16,60,61]. In this study, microbubbles demonstrated successful enhancement at a frequency of 3.5 MHz in a 0.3 T MRI system. Figure 10 shows the ultrasound images of water, unloaded PCNDs and 10 mg loaded SPION-loaded PCNDs taken at a frequency of 3.5 MHz with MI varying from 0.1 to 1.0.

In the current study, a 10 mg concentration of SPIONs gave the best response in 3.5 MHz ultrasound and also an acceptable response in MRI with a decrease in intensity up to 32.2 AU from 1200 AU of water (Figure 7). Therefore, this concentration was considered as the optimum one, and all safety assays were then performed at this concentration. Safety assays were also performed on unloaded PCNDs for comparison and for their excellent potential to be used as ultrasound contrast agents alone and for dual-mode imaging of ultrasound and T1 weighted MRI.

### 3.7. Stability Testing

**Figure 11:**
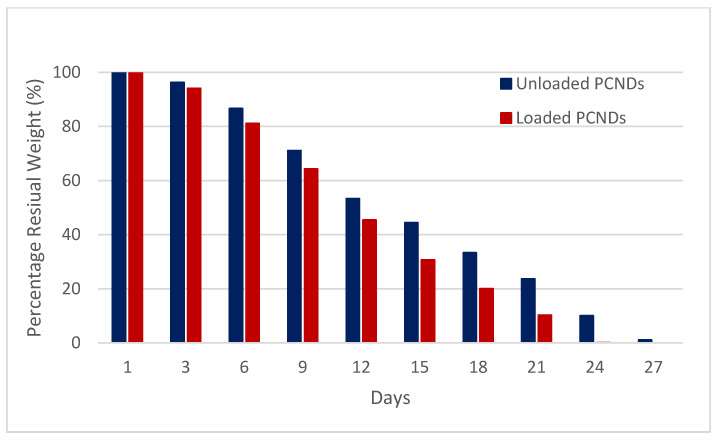
Degradation profile of PCNDs shows loaded PCNDs degrading faster than unloaded PCNDs.

Both non-suspended unloaded and loaded PCNDs showed no mass loss and similar contrast levels upon ultrasonography when stored at room temperature 25 °C, even after 6 months, while PCNDs suspended in PBS buffer and incubated at 37 °C, mimicking body temperature, showed complete degradation in 27 days. The degradability of loaded PCNDs was faster than unloaded PCNDs with complete degradation in 21 days (Figure 11).

### 3.8. PCNDs Safety Analysis

Systemically administered nanoparticles first came into contact with blood cells where they can disturb hemostatic and hemodynamic functions. Studies have also reported some serious complications as a result of intravenous administration including hemorrhagic and thrombotic complications. Therefore, tests for coagulation profile, thrombosis, hemolysis and complement activation as recommended by ISO-10993-4 for medical devices interacting with blood should also be performed for intravenously administered nanoparticles [62]. For establishing the safety of our PCNDs, hemocompatibility tests were performed. PCNDs were found to be safe as they did not trigger or delay any coagulation cascade nor cause any damage to RBCs. PCNDs showed a thrombin time of 9 s and plasma recalcification time of 240 s, which shows that PCNDs have no effect on bleeding time and did not affect the clotting process at any stage (Table 4).

**Figure 12:**
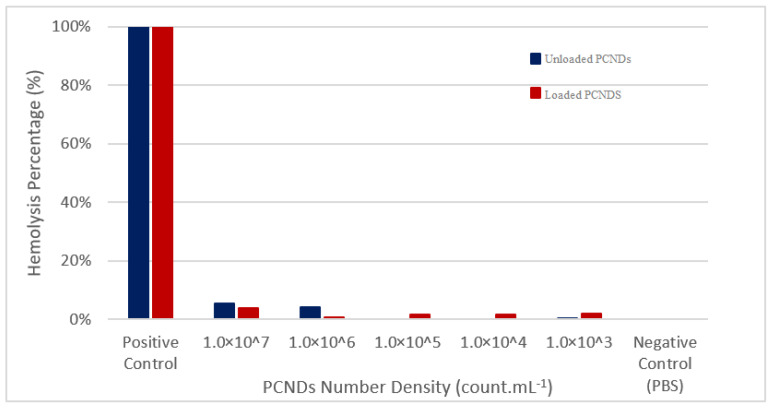
Hemolysis assay showing less than 6% hemolysis at all concentrations.

A hemolysis assay was also performed to check PCND’s interaction with red blood cells. Even the highest concentration (1 × 10^7^ PCNDs) showed less than 6% hemolysis which is considered negligible according to ISO standards [63] (Figure 12). These assays showed good hemocompatibility of both unloaded and loaded multimodal contrast agents.

**Figure 13:**
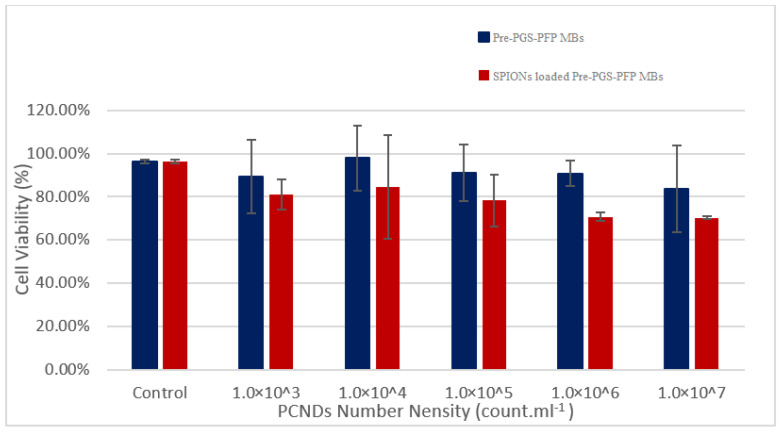
Results of the MTT assay showing cell viability of both constructs to be more than 70% which is a safety requirement by ISO for approval of any biomedical material.

The fully cured PGS polymer has excellent biocompatibility and is approved by the FDA for medical applications. However, very little data are available about the safety of partially cured pre-PGS polymer. Therefore, we performed an MTT assay for biocompatibility on triple-negative breast cancer MCF-7 cell lines. The unloaded PCNDs showed more than 80% viability (Figure 13) at all doses which proves their safety and negligible cytotoxic effects on cells. SPION-loaded PCNDs showed low viability compared to unloaded PCNDs; however, it was still under the ISO approved limit (i.e., at least 70% viability) to be used for biomedical purposes [64]. The reason for low viability could be due to the effect of the redox-active surface of SPIONs on mitochondrial activity or due to interference of PEG-coated SPIONs absorbance at 550 nm as explained by previous research [65,66,67,68].

One of the serious risks associated with nanomedicines is an activation of the immune system upon drug administration. According to literature, around 50% of the nanomaterials trigger the immune response in vivo upon administration. Among them, complement activation-related pseudo allergic reactions (CARPA) is the most common type of immune reaction [69]. CARPA reactions have been also noted with clinically approve nanomedicines such as Doxil, Taxol, and Feraheme. All of them are now sold with black boxes warning of potentially life-threatening immune reactions [70]. The risk of CARPA was also found to be associated with available marketed ultrasound contrast agents such as Definity and Optison which, after four deaths due to severe cardiopulmonary distress in 2007, are sold with a black box warning [71]. Therefore, in vitro analysis of the immune response to these drugs is necessary. The most commonly used technique for predicting CARPA in vitro is the sandwich ELISA technique for the detection of all the complement factors C3–C5 [72]. To our knowledge, pre-PGS has never been employed in contrast agent designing; therefore, we performed ELISA for C5b complement protein. We choose the C5b fragment due to its negligible amount under normal conditions. It is only formed as a result of complement cascade activating C3 which leads to the formation of various bioactive molecules including C5a and C5b. C5b is the first complement component that leads to membrane attack complex (MAC) formation which is an important immune effector [73]. The results of the current ELISA showed both loaded and unloaded PCNDs did not result in C5b formation while a positive control resulted in 24.6 µg/L of C5b, which shows that both PCNDs are non-immunogenic and safe to administer (Supplementary Figure S3).

## 4. Conclusions

PEG-coated SPION-loaded PCNDs show excellent potential to be used as multimodal contrast agents for both MRI and ultrasound. PCNDs with 10 mg nanoparticle concentration showed excellent enhancement in ultrasound, a high susceptibility effect on T2 weighted MR images and good hemodynamic compatibility without eliciting any immune response. Another interesting finding was the multimodal potential of unloaded pre-PGS shell-based PCNDs as T1 MR contrast agent and ultrasound contrast agent which needs to be further explicated.

## Figures and Tables

**Table 1:** FTIR peaks and their related functional groups.

Functional Group	Wavenumber (cm^−1^)	References
C-F stretch	1100–1300	[24,25]
Fe-O Stretching	630, 795, 880, 2920	[26,27,28,29]
C=O stretch	1735–1760	[30,31]
C-H stretch	2800–2900	[32]
C-H bending	600–900	[32]

**Table 2:** XRD peaks observed in current study and references for similar peaks in other studies.

Constructs	2θ Peaks Observed (°)	Reference
Magnetite	31.35, 35.35, 45.1, 57.15, 63.07 and 74.5	[33,35,36]
Pre-PGS	25° and 35°	[22,34]

**Table 3:** Morphological characterization of PEG-coated iron oxide nanoparticles and PEG-coated iron oxide loaded PCNDs and unloaded PCNDs.

Suspensions	SEM Avg Size (nm)	DLS Avg Size (nm)	Zeta Potential (mV)
PEG-coated SPIONs	173 ± 29	190.1 ± 0.0	+32 ± 7
Unloaded PCNDs (non-converted)	132 ± 58	210 ± 61	−21 ± 4
PEG-coated SPION-loaded PCNDs (non-converted)	156 ± 95	164 ± 26	−12 ± 5
Unloaded PCNDs (phase-converted)		1076 ± 288 5359 ± 342	−21 ± 4
PEG-coated SPION-loaded PCNDs (phase-converted)		927 ± 167	−12 ± 5

**Table 4:** Bleeding time assays.

Concentrations	Average Thrombin Time (s)		Plasma Recalcification Time (s)	
	Unloaded PCNDs	10 mg SPIONs Loaded PCNDs	Unloaded PCNDs	10 mg SPIONs Loaded PCNDs
Plasma	8	8	240	240
Plasma with PBS	10	10	220	220
PCNDs (1 × 10^7^)	13	9	210	240
PCNDs (1 × 10^5^)	10	6	210	245
PCNDs (1 × 10^3^)	10	6	210	250
Aspirin	35	35	410	410
Normal Time	<21	<21	220–350	220–350

## Data Availability

The authors declare that they have no known competing financial interests or personal relationships that could have appeared to influence the work reported in this paper.

## Supplementary Materials

The following supporting information can be downloaded at: https://www.mdpi.com/article/10.3390/polym14142915/s1. Figure S1 showing calibration curve and formula for calculating encapsulation efficiency of PCNDs; Figure S2. showing roughness plot on unloaded PCNDs (a), iron oxide nanoparticle (b) and loaded PCNDs (c); Figure S3. Results of ELISA for detecting C5b showing non-activation for PCNDs and negative control while positive control having C5b levels of 24.6 µg/L; Table S1 showing roughness measurements calculated from surface plots; Table S2: ANOVA analysis on T2 image intensities of construct and positive control (PC) and negative control (NC); Table S3: ANOVA analysis on T1 image intensities of construct and positive control (PC) and negative control (NC).
