# Supplementary material for: Novel Magnetic Elastic Phase-Change Nanodroplets as Dual Mode Contrast Agent for Ultrasound and Magnetic Resonance Imaging
Source: Polymers (Basel). 2022 Jul 19;14(14):2915. doi: 10.3390/polym14142915 (PMC9318938; doi:10.3390/polym14142915)
Supplement: Supplementary file 1 [file polymers-14-02915-s001.zip › polymers-1718245-supplementary.pdf]

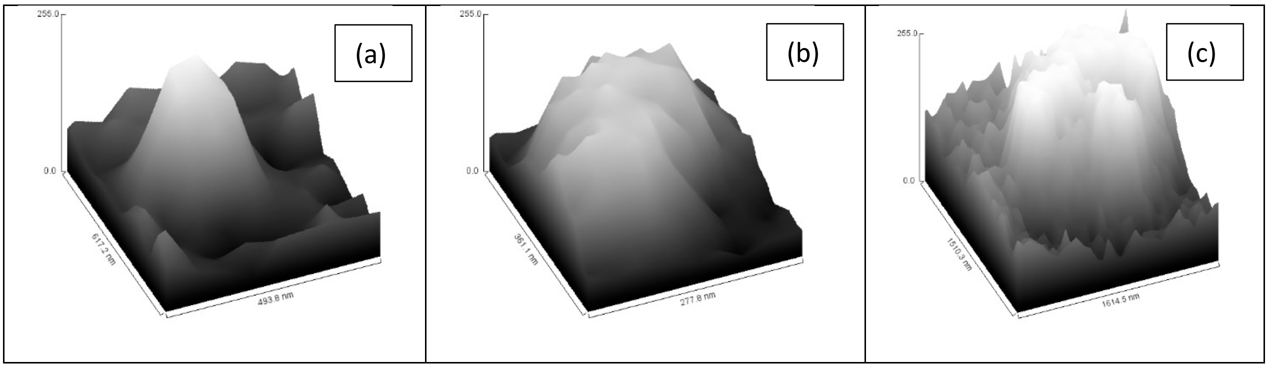

Supplementary Figure S1 showing roughness plot on unloaded PCNDs (a), iron oxide nanoparticle (b) and loaded PCNDs (c).

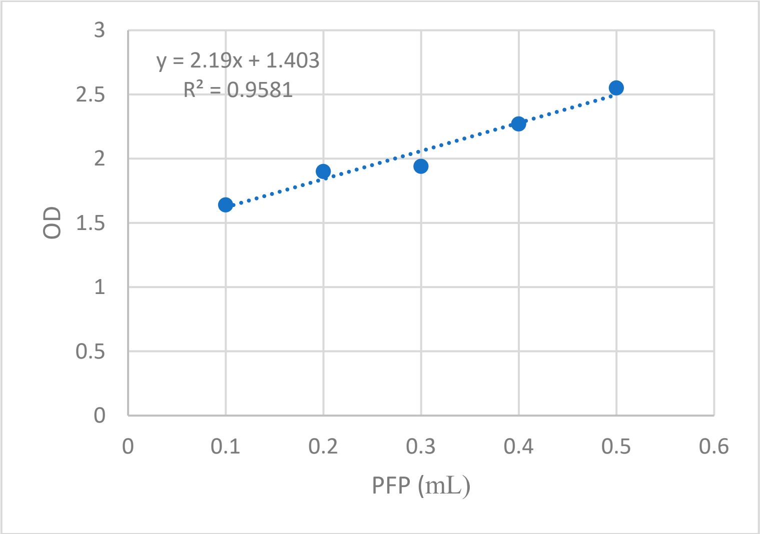

(1.98-1.403)/2.19= 0.2637

EE (%)=0.2637*100/0.5=52.6%

Supplementary Figure S2 showing calibration curve and formula for calculating encapsulation efficiency of PCNDs.

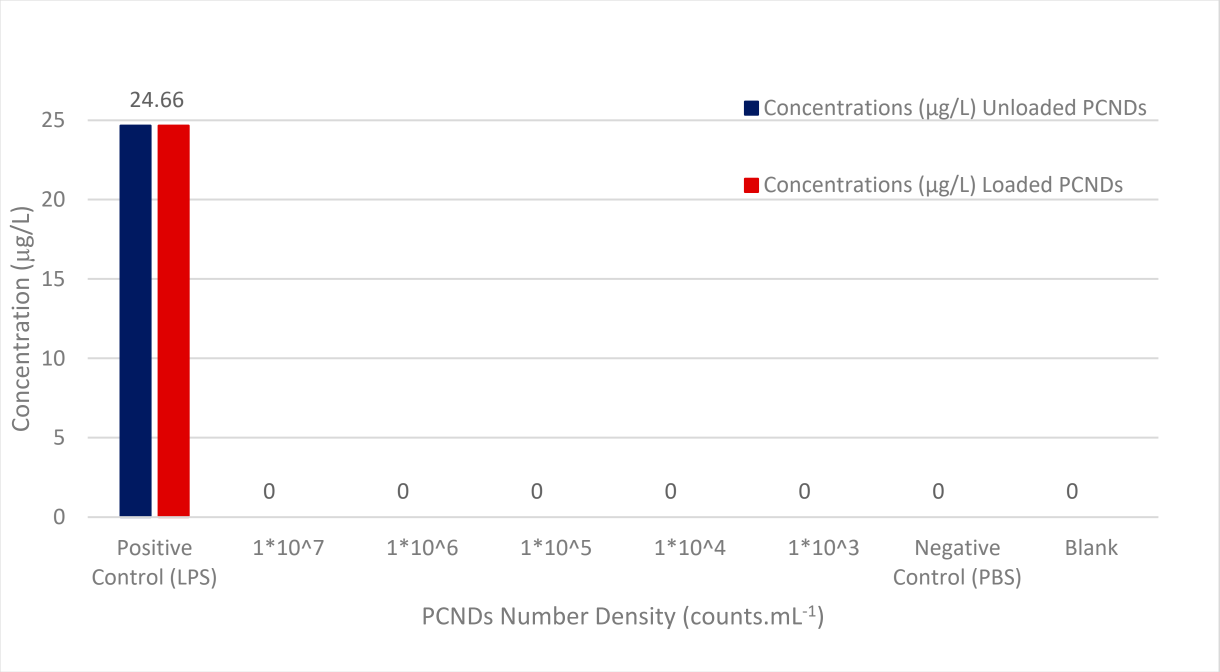

Supplementary Figure S3. Results of ELISA for detecting C5b showing non-activation for PCNDs and negative control while positive control having C5b levels of 24.6 µg/L.

Supplementary Table S1 showing roughness measurements calculated from surface plots.

	Root Mean Square Roughness (Rq) (nm)	Average Roughness (Ra) (nm)
Unloaded PCNDs	200.482	176.611
Iron oxide NPs	207.727	188.993
Loaded PCNDs	215.257	201.461

Supplementary Table S2: ANOVA analysis on T2 image intensities of construct and positive control (PC) and negative control (NC).

Multiple comparisons test	Mean Diff.	95.00% CI of diff.	Significant?	Summary	Adjusted P Value	
NC Water vs. SPIONs (PC)	1296	1220 to 1372	Yes	****	<0.0001	A-B
NC Water vs. Unloaded MB	951.0	875.1 to 1027	Yes	****	<0.0001	A-C
NC Water vs. Loaded MB (shell)	1259	1183 to 1335	Yes	****	<0.0001	A-D
NC Water vs. Gadovist	1291	1215 to 1367	Yes	****	<0.0001	A-E
SPIONs (PC) vs. Unloaded MB	-344.9	-420.9 to -269.0	Yes	****	<0.0001	B-C
SPIONs (PC) vs. Loaded MB (shell)	-36.82	-112.8 to 39.16	No	ns	0.5318	B-D
SPIONs (PC) vs. Gadovist	-5.200	-81.18 to 70.78	No	ns	0.9993	B-E
Unloaded MB vs. Loaded MB (shell)	308.1	232.1 to 384.1	Yes	****	<0.0001	C-D
Unloaded MB vs. Gadovist	339.7	263.8 to 415.7	Yes	****	<0.0001	C-E
Loaded MB (shell) vs. Gadovist	31.62	-44.36 to 107.6	No	ns	0.6584	D-E

Supplementary Table S3: ANOVA analysis on T1 image intensities of construct and positive control (PC) and negative control (NC).

Multiple comparisons test	Mean Diff.	95.00% CI of diff.	Significant?	Summary	Adjusted P Value	
NC Water vs. SPIONs	454.5	96.42 to 812.6	Yes	*	0.0109	A-B
NC Water vs. Unloaded MB	-702.5	-1061 to -344.4	Yes	***	0.0004	A-C
NC Water vs. Loaded MB (shell)	27.37	-330.7 to 385.4	No	ns	>0.9999	A-D
NC Water vs. Gadovist	-822.2	-1180 to -464.1	Yes	****	<0.0001	A-E
SPIONs vs. Unloaded MB	-1157	-1515 to -798.9	Yes	****	<0.0001	B-C
SPIONs vs. Loaded MB (shell)	-427.1	-785.2 to -69.06	Yes	*	0.0166	B-D
SPIONs vs. Gadovist	-1277	-1635 to -918.6	Yes	****	<0.0001	B-E
Unloaded MB vs. Loaded MB (shell)	729.9	371.8 to 1088	Yes	***	0.0003	C-D
Unloaded MB vs. Gadovist (PC)	-119.7	-477.8 to 238.4	No	ns	0.9511	C-E
Loaded MB (shell) vs. Gadovist (PC)	-849.6	-1208 to -491.5	Yes	****	<0.0001	D-E
